# Family-Centered Care Experiences in Elderly with Chronic Diseases in Communities: Qualitative Study of Patients, Families, Nurses, and Volunteers

**DOI:** 10.1089/heq.2024.0009

**Published:** 2024-06-05

**Authors:** Ah Yusuf, Jenny Marlindawani Purba, Dewi Eka Putri, Ronal Surya Aditya, Abdullah Saleh Alruwaili, Daifallah M. AlRazeeni

**Affiliations:** ^1^ Faculty of Nursing, Universitas Airlangga, Surabaya, Indonesia.; ^2^ Dimentia and Aging Care Research Center, Universitas Airlangga, Indonesia.; ^3^ Community and Psychiatric Department, Faculty of Nursing, Universitas Sumatera Utara, Medan, Indonesia.; ^4^ Community and Psychiatric Department, Faculty of Nursing, Universitas Andalas, Padang, Indonesia.; ^5^ Department of Nursing, Faculty of Medicine, Universitas Negeri, Malang, Indonesia.; ^6^ Emergency Medical Services Program, College of Applied Medical Sciences, King Saud Bin Abdulaziz University for Health Sciences, Al Ahsa, Saudi Arabia.; ^7^ King Abdullah International Medical Research Center, Al Ahsa, Saudi Arabia.; ^8^ School of Health, University of New England, Armidale, Australia.; ^9^ Department of EMS, King Saud University (KSU), Riyadh, Saudi Arabia.

**Keywords:** Humans, Aged, Quality of life, Focus groups, Parents, Volunteers, Good health and well-being

## Abstract

**Introduction:**

The family, at community, must be an important part of elderly care. However, most of the elderly experience a decrease in psychological well-being and quality of life. This is not in accordance with the concept of the family-centered care (FCC) model and can endanger the continuity of the elderly with chronic illnesses.

**Objective:**

The aim of this study was to explore experience of FCC among patients with chronic illness, nurses, families, and volunteers.

**Methods:**

This study examines the contextual-based FCC model qualitatively. In-depth interviews and focus group discussions were conducted by 12 elderly people, 3 nurses, 10 family members, and 3 volunteers. In total, 36 people, where several were FGD participants, were also interviewed in depth. Data were analyzed using thematic analysis, with codes organized into larger themes.

**Result:**

In total, 36 interviews and FGDs were conducted. The concept of information interaction, the phenomenon of emotional interaction, the practical interaction, and various factors that can either facilitate or impede interaction, were considered the four significant themes.

**Conclusion:**

FCC acceptance is found in interactions between parents, nurses, family, and volunteers who are not optimistic. Lack of communication and collaboration was noted between community nurses and volunteers. Offer a new perspective on developing and implementing interventions that facilitate positive interactions, reduce family burdens, provide high-quality treatment for parents with chronic diseases, and improve the quality of care for those with advanced chronic conditions.

**Recommendation:**

These insights provide a fresh perspective on how to develop and implement effective interventions in this context. It is recommended that future research should employ multiple methodologies to investigate FCC across diverse health practices for the elderly population.

## Introduction

The prevalence of chronic disease on a global scale is on the rise. It is projected that by the year 2021, the prevalence rate among the global population will rise to 57%, resulting in 75% of all fatalities worldwide.^1^ To address the negative health outcomes and enhance overall well-being, it is imperative to develop novel approaches to health care delivery. The implementation of a chronic care model can be considered an innovative approach to promoting self-care. As many as 24.6% of the elderly population (elderly) in Indonesia have a history of chronic disease. Of the elderly group with this history, the majority or 37.8% had hypertension. In addition, 22.9% had diabetes, 11.9% rheumatic disease, and 11.4% had heart disease. The efficacy of chronic patient management may be constrained by inadequate comprehension among health care providers of the intricate cultural milieu, coupled with a lack of acknowledgment of the substantial impact of familial dynamics on individual health conduct.^2^ The patient’s cultural identity and social network encompass family members, whose impact on the patient’s self-care can be either positive or negative. To facilitate the provision of suitable self-care assistance by families, it is imperative to encourage, facilitate, and direct their participation in the care of individuals with persistent medical conditions.

The emphasis on factors that promote self-care can be referred to as a patient-centered or family-centered approach. The terms patient-centered care and family-centered care (FCC) have been utilized interchangeably or in conjunction with one another. However, there are those who have construed the former to denote care that is focused solely on the patient. While acknowledging the importance of the patient’s family, the concept of “patient-focused care” prioritizes the patient’s individual preferences and values in the context of the consultation. In contrast, FCC is characterized as a care approach that takes into account the requirements of both the patient and their family. Moreover, it has been conceptualized as a health care delivery strategy that endows the family with the capacity to act as a collaborator in the provision of care for a particular individual.^2^ When patients and their families are regarded as collaborators with the primary care physician, they can engage in the process of diagnosis and treatment decision-making.^3^

Various theoretical frameworks have been employed to delineate the potential methods by which the activities of the FCC may be executed.^4^ The consultative process is characterized by collaborative partnerships, joint decision-making, and shared leadership.^5^ During the consultation, self-care and family care were discussed by other individuals. Cole-Kelly et al. and McDaniel et al. have outlined effective strategies for implementing family-centered consultations in clinical settings and have also proposed a straightforward method for demonstrating the role of families in care decision-making, particularly in the context of direct patient care. The methodology outlined by Cole-Kelly entails inquiries regarding the patient’s familial medical history and their perspective on how their family can assist in managing their health concerns.^6^ McDaniel’s characterization of family involvement spans from limited participation of older family members in the caregiving process to the provision of family therapy.^7^ Psychological well-being and quality of life are both necessary factors for reducing depression rates among the elderly. Along with psychological well-being, the quality of life and happiness among the elderly improve if they are cared for in their own homes and with their own families.

The concept of FCC has gained global recognition as a fundamental component of the chronic care model.^8^ The benefits of FCC have been a focal point of arguments in its favor, with emphasis placed on its potential to promote equity in the delivery of health care, enhance patient safety, and improve the overall quality of care. Additional advantages that have been reported encompass decreased health care expenses for both the patient and the health care institution, enhancement of patient contentment, and adherence to clinical management protocols.^9^ Based on previous research, no direct information is available regarding the limitations of FCC experiences in older patients with chronic illnesses in the community. However, we can mention that previous research provides insight into the experiences and needs of family caregivers for older adults with chronic illnesses, highlighting the burdens and challenges they face.^10–15^ Thus, this does not directly address the limited experience of FCC for elderly patients with chronic illnesses in the community.

In the ASEAN context, particularly in Indonesia, there appears to be a scarcity of literature elucidating FCC concepts and practices in comparison to developed nations. The extant literature indicates that conducting regular family-oriented interviews can enhance the perceived family functioning among elderly individuals receiving care in Indonesian communities.^16^ According to a study conducted in Malawi, the scarcity of health care personnel necessitated the involvement of family members in the provision of hospital care for their patients. Research conducted in Lesotho and Mozambique has revealed that volunteers such as parents and other family members are frequently marginalized from the care process as a result of communication challenges with health care providers responsible for treating their patients.^17^ Currently, a search was conducted on PubMed and Google Scholar utilizing the MeSH terms or keywords “Asia,” “patient perception,” “family-centered care,” and “chronic disease,” which yielded no pertinent findings.

Before adapting and implementing the FCC in an Asian context, it is very important to first gain experience of seniors, families, volunteers, and volunteers related to the FCC.^18^ This step is considered important in promoting the FCC in the Asian context. Qualitative research was chosen because these experiences have the potential to facilitate understanding of the aging population’s trends and willingness to embrace the FCC in that setting. The primary aim of this investigation was to examine the perspectives of community-dwelling elderly people with chronic illness regarding FCC. The aim of this study was to investigate FCC experiences among patients with chronic illness, caregivers, families, and volunteers.

## Methods

### Study design

The study employed a qualitative exploratory descriptive design to investigate the contextual practices of FCC among elderly individuals, families, nurses, and volunteers in the community.^19^ The application of a qualitative exploratory descriptive design in this research is to highlight the use of thematic analysis and exploratory mapping in the context of patients, families, nurses, and volunteers. The rationale behind the implementation of this particular design is to provide an elucidation of the FCC phenomenon through the lenses of the elderly, families, volunteers, and volunteers within the community. Furthermore, the theoretical framework employed is the FCC. This presents a chance to examine the application of FCC within a community care facility situated in Indonesia. The study was carried out during the period spanning from June to August 2022.

This research has the four criteria of rigor which consists of credibility, transferability, dependability, and confirmability. Credibility of this research. i.e., use of appropriate methodology, such as collecting data directly from relevant participants (patients, families, nurses, and volunteers).^20^ Transferability of this research provides a detailed description of research procedures, including data collection and analysis methods.^21^ The dependability of this research is to describe in detail the methodological steps taken in the research, from research design to data analysis.^22^ Confirmability of this research is separating the researcher from the data collected, thereby minimizing researcher bias.^23^

### Setting

The study took place in a community center in Medan City Indonesia. To develop FCC model for elderly with chronic illness, a purposive sampling technique was made to selected participants. Participants’ and stakeholders’ willingness to engage in the research influenced the selection of this study’s location. In addition, the number of elderly people suffering from chronic illness is one of the highest in the city of Medan.^24^

### Participant

**FIG. 1.**
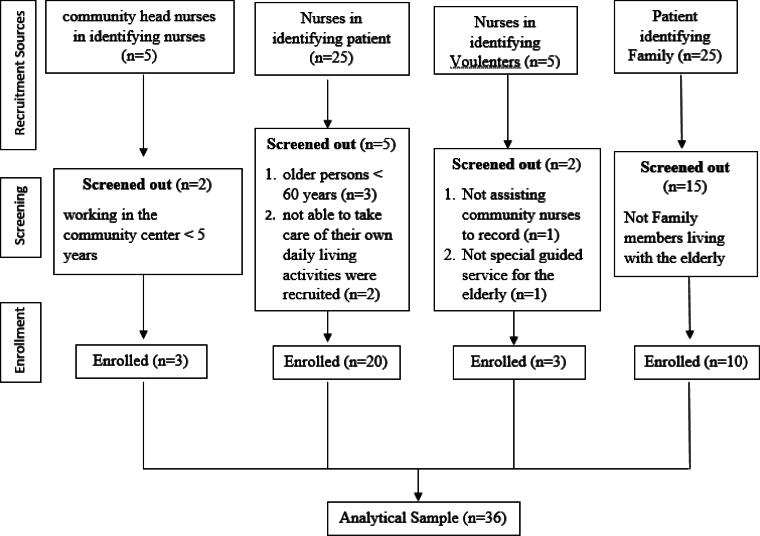
Flow diagram of participants (*n* = 36).

In total, there were 36 respondents in the study including elderly with chronic illness (*n* = 20), family (*n* = 10), nurses (*n* = 3), and volunteers (*n* = 3) (Fig. 1). Indonesian older persons aged >60 years and able to take care of their own daily living activities were recruited. Family members living with the elderly also participated in this study. Three community nurses became participants. All of them have been working in the community center for >5 years. The community health volunteers (cadres) assisted community nurses in recording the health status of elderly individuals who participated in a special guided program.

### Data collection

Ethics approvals and cover letters were used to seek authorization from department and unit managers in two community settings. Researchers were assisted by community head nurses in identifying nurses who met the study inclusion criteria. The head nurse makes similar provisions for household groups residing in the Medan area. After that, study participants were given an information sheet and they provided informed consent, which included additional consent for voice recordings, if they agreed to take part in the study. To achieve data triangulation and collect multiple perspectives from participants, researchers used interviews and focus group discussions (FGDs) as data collection methods. The lead researcher conducted interviews and FGDs with the help of six skilled research assistants. This study used interviews and FGD which were conducted in a safe place at each location, facilitated by the community head nurse. Interviewers used open-ended questions with follow-up questions to gain detailed insight, and they kept track of the issues discussed. It is very important to prevent potential interference resulting from recorded data. Investigators deliberately selected a sample of 36 people to take part in one-on-one interviews and group discussions. The concept explored in the interviews that became the main topic of the “Implementation of the Family-Centered Care (FCC) Model” research were:

1. What are the experiences implementing the family-centered care (FCC) model of care?
2. What is the phenomena that occurred during the family-centered care (FCC) implementation?
3. What are the barriers and supporters of family-centered care (FCC) implementation?
4. How do older patients with chronic illnesses experience receiving family-centered care in a community setting?
5. What are families’ perceptions and expectations of family-centered care for elderly family members suffering from chronic illnesses?
6. What is the role and experience of nurses in supporting the implementation of the family-centered care (FCC) model for elderly patients with chronic diseases in the community?
7. How do volunteers in the community contribute to supporting families and older patients with chronic illnesses in the context of the FCC model?
8. How do interactions and collaboration between patients, families, caregivers, and volunteers influence the experiences and outcomes of care for older patients with chronic illnesses in the community?
9. How do social, cultural, and economic factors influence the implementation of the FCC model in caring for elderly patients with chronic illnesses in community settings?
10. How are elderly patients with chronic diseases involved in decisions regarding their care in the context of the FCC model?
11. What are the challenges faced by care providers (families, nurses, and volunteers) in implementing the FCC model for elderly patients with chronic diseases in the community?
12. How do patients, families, and caregivers feel satisfied and engaged with FCC for older patients with chronic illnesses?
13. How can support from community organizations and structures facilitate or hinder implementation of the FCC model for older patients with chronic illnesses in the community?

FGD with a minimum duration of 45 min. Each interview is subject to a minimum of 30 min of individual questions. The rationale behind the collection of diverse data forms is to obtain rich data which can offer a comprehensive understanding of the FCC phenomenon within the community. Table 1 provides additional information on the qualitative data collected for the current investigation.

**Table 1.** Demographic Data of the Nurses, Families, and Volunteers

No	Respondent	Variable	*n*
1	Nurse	Education	
		Vocational	2
		Undergraduate	1
		Gender	
		Male	1
		Female	2
		Experience	
		0–5 Years	1
		6–10 Years	2
2	Family	Education	
		Junior High School	8
		Senior High School	2
		Gender	
		Male	2
		Female	8
3	Volunteers	Education	
		Junior High School	2
		Senior High School	1
		Gender	
		Male	0
		Female	3
		Experience	
		0–5 Years	2
		6–10 Years	1

### Data analysis

The researchers transcribed all data into English text with the assistance of a linguist, given that the interview language utilized by the participants was the local dialect (Medan).^25^ The researchers thoroughly examined the transcripts multiple times to ensure grammatical accuracy and to fully engage with the data. To maintain the integrity of anonymity and confidentiality, codes were affixed to transcripts, settings, and participants.^26^ The present investigation involved the coding of interviews as “I,” focus group discussions as “D,” older as “O,” families as “F,” volunteers as “R,” and nurses as “C.”^27^ The addition of serial numbers to the aforementioned codes resulted in the creation of anonymous codes. The investigators employed an open inductive content analysis technique to extract fragmented data from the transcripts.^28^ This involved comparing the transcripts of the data and scrutinizing their similarities, differences, and patterns.^29^ The primary code was maintained in close proximity to the language used by the participants, with the aim of upholding methodological rigor. Subsequently, the scholars employed a method known as focused coding to enhance and theoretically sample the data, which was categorized into groups by selecting pivotal terms that classify the preliminary code generated from the data.^30^ Ultimately, the researchers engaged in a process of reflection regarding the emergent categories of data, with the aim of integrating and synthesizing the fragmented data to gain a comprehensive understanding of participants’ contextual perspectives on the practice of the Federal Communications Commission within the community. The process of iterative content analysis was executed by importing all 36 transcripts into the NVIVO qualitative software. The analysis was conducted using qualitative software tools such as lexical search, word cloud, comparison of retrieval patterns, and code/sub-code model functions. The completeness and validity of the analysis were assessed using the Consolidated Criteria for Qualitative Research Reporting.^31^

#### Ethical considerations

This study received ethical approval (No.: 2738/VII/SP/2022). All participants received written and verbal explanation about the research objectives and process. The data collected is kept confidential and participants can withdraw from the research process at any time without being penalized. Those agreeing to participate in this study were asked to sign an informed consent form.

## Result

The results of the study are presented in several tables: (Table 1) Demographic data of the nurse, family, and community health volunteers. (Table 2) Themes and sub-themes.

**Table 2.** Theme and Sub-Themes

Theme	Sub-theme
The concept of information interaction.	a. The establishment of a positive interactive relationship is predicated upon mutual communication
	b. The foundation of information interaction is the sharing of information
The phenomenon of emotional interaction.	a. The perception of positive emotional interaction is crucial
	b. The perception of negative emotional interaction is significant
The practical interaction	a. The provision of medical treatment assistance is essential
	b. The management of health for elderly individuals with disabilities is critical
Various factors can either facilitate or impede interaction.	a. Factors that facilitate interaction
	b. Factors that impede interaction

### Theme 1: The Concept of Information Interaction

#### The establishment of a positive interactive relationship is predicated upon mutual communication

Communication serves as an intermediary between the sender and recipient, and is a fundamental mechanism underlying human conduct. In both verbal and nonverbal communication, individuals transmit information, interpret the meaning of the information, and adapt their behaviors based on that meaning and the surrounding context. Individuals who engage in communication with one another have a reciprocal impact on each other through an ongoing exchange of feedback. The phenomenon of positive circular pattern of communication is observed when two communicators engage in harmonious cooperation. The act of observing interactions and interdependence among family members and between nurses and family members has been deemed useful in literature.

The temporal extent of the services rendered by community nurses on each occasion was considerably restricted, thereby impeding the possibility of engaging in thorough communication. Despite their desire to obtain information from medical personnel, they refrained from communicating with community nurses owing to apprehension about causing disruption or inconvenience.

*O1, O3, O5, F2, F3: “It appears that there is a significant amount of tasks that they need to accomplish. They depart shortly after posing a limited number of inquiries. The individuals in question are currently unavailable to engage in conversation, however, I am in dire need of assistance and pertinent information. I earnestly desire for an individual to allocate additional time towards elucidating the subject matter to me”.*

*O4, O6, O7, F1, F5: “It is acknowledged that individuals lead busy lives. In general, the speaker expresses a desire to avoid causing inconvenience or difficulty for others. Occasionally, when circumstances compel me, I proactively undertake the action of initiating a telephone conversation with them”.*

Currently, the mode of communication among the elderly, families, nurses, and volunteers primarily involves telephonic assistance. Volunteers aspire to explore diverse communication channels with nurses to facilitate efficient communication. The majority of families and volunteers express a desire to enhance the frequency of their communication with health care professionals, particularly nurses.

*O8, O10, O11, F7: “Occasionally, individuals make inquiries regarding the well-being of their progenitors. Increased community visitation or a higher frequency of community lectures would enhance the efficacy of face-to-face communication.”*

*O9, O12, 016, F8, F9: “My interactions with community nurses are limited, and their presence in this location is infrequent. If only they could frequent this location.”*

#### The foundation of information interaction is the sharing of information

The individuals responsible for providing care to the elderly, including volunteers, family members, nurses, and volunteers, possess a high level of expertise within their respective domains. The reciprocal relationship is characterized by equivalence. The elderly and their families possess a wealth of expertise regarding their own lives and are well-versed in the intricacies of the disease. In contrast, nurses and volunteers possess expertise in comprehending the encounter with illness owing to their educational background and practical training. Effective measures entail providing holistic care to parents with chronic illnesses, which encompasses addressing the concerns of their family members by disseminating relevant information and responding to their queries. The act of sharing information and experiences has a positive impact on the provision of care for parents who are afflicted with chronic illnesses. The majority of family members caring for parents with chronic illnesses lack medical training and seek to acquire additional knowledge regarding home-based care. The adequacy of information presented should align with the specific requirements of each volunteer. The volunteers aspire to acquire knowledge pertaining to maintenance, while also seeking to establish a platform for the exchange of information that can be utilized for health consultation purposes.

*R1, R2, F1, F3: “I provide in-home care for elderly individuals. I require further information regarding the actions that are within my purview and those that are not”.*

*R3, F5, F7: “It is suggested that written material or video resources be utilized instead of relying solely on oral communication. Additionally, it is recommended that the use of professional jargon be minimized in order to facilitate comprehension, particularly for older individuals.”*

*R2, F8, F9, F10:“It is my belief that we are capable of effectively administering the WhatsApp group. In the event of encountering difficulties, it is possible to seek consultation, which is tantamount to engaging in resource-sharing. In my opinion, this is of high quality.”*

### Theme 3: The Phenomenon of Emotional Interaction

#### The perception of positive emotional interaction is crucial

Positive experiences can be fostered through effective interaction among elderly individuals, their family members, nurses, and volunteers. The elderly, their family members, and volunteers seek to establish communication with a broad range of community nurses regarding this ailment. Upon receiving recognition and commendation from nurses, volunteers may gain recognition and esteem, ultimately contributing to the provision of improved care for elderly individuals afflicted with chronic illnesses.

*R1, F1, F4, F6: “The nurse in question is highly skilled and proficient in their duties. He frequently inquires about certain illnesses via telephone. The individual in question exhibits a positive attitude and expresses gratitude towards the subject in question.”*

*R2, F2, F7: “Engaging with nurses is a beneficial practice, as their support and motivation can enhance one’s confidence in the treatment process.”*

*R3, F3, F5, F8: “At a prior instance, during a visit by a nurse to replace the gastric tube, commendation was also given to me for providing adequate care for my spouse. The author experiences a strong sense of happiness and pride.”*

#### The perception of negative emotional interaction is significant

Nonetheless, according to numerous volunteers, community nurses are presently prioritizing parents with chronic ailments, while neglecting to provide adequate attention to families and volunteers. Because of prolonged exposure to high levels of psychological stress, both family members and volunteers are experiencing significant psychological distress that cannot be easily alleviated. Consequently, it is imperative that family members and volunteers receive psychological counseling to mitigate the burden of caregiving and sustain their positive emotional well-being.

*“It is commonly perceived that there exists a certain expectation of actions that I am required to undertake. There is no necessity to provide care for my father. The healthcare professional fails to provide me with adequate attention. As a result of lacking patience, I am unable to endure extended periods of time.”*

*“In certain instances, when engaging in conversations with nurses, there may be a perceived display of haughtiness and aloofness in their demeanor. The emotional response elicited is that of sadness.”*

*“The individual expresses a lack of sufficient rest owing to their responsibility of caring for their spouse, which elicits feelings of distress. The nurse consistently exhibits a deficiency in acknowledging my efforts, resulting in heightened levels of stress and a sense of inadequacy.”*

### Theme 3: The Practical Interaction

#### The provision of medical treatment assistance is essential

The health care needs of parents with chronic illnesses necessitate their attendance at appropriate medical facilities. However, as a result of limited opportunities for external communication, families with disabled elderly members experience chronic social isolation and illness. Second, it is advisable for families to take a disciplined approach toward accompanying parents with varying interests to health care facilities. Individuals encounter physical functional constraints and environmental challenges that impede their abilities. Hence, the process of accessing health care services is a subject of variation for both households and volunteers, necessitating the coordination of health service visits by nurses.

*F1, F3, F5, R1: “In instances where my paternal figure experiences physical discomfort. The act of taking him out elicits discomfort. Our residence is characterized by a limited width. My initial point of contact for assistance is typically the nurse, as we maintain a close relationship.”*

*F2, F4, F6, R2: “In the event that the patient requires medical attention, it is possible to arrange for a physician to conduct an initial diagnosis at the patient's doorstep to determine whether hospitalization is necessary. This action prevents him from throwing back.”*

*F7, F9, R3: “The duration of hospitalization for inpatients in large hospitals is currently brief. In order to sustain hospitalization and conditioning, relocation to a hospital facility is imperative, albeit a cumbersome process.”*

#### The management of health for elderly individuals with chronic illnesses is critical

Parents who are afflicted with chronic illnesses over extended periods of time require long-term care. The practice of monitoring various health indicators, such as blood pressure, blood glucose levels, and vital signs, is a crucial aspect of health care management. However, certain families may lack the necessary skills or knowledge to perform these tasks effectively. A significant proportion of family members lack prior experience in administering medical treatment and may experience confusion when confronted with the complexities of patient illnesses and disease-related complications. The individuals aspire to receive nursing guidance and support pertaining to rehabilitation exercises for parents with chronic disease. The elderly and their families face limitations in accessing adequate assistance and resources from nurses and volunteers. There is a lack of collaboration between nurses and volunteers, as well as between the elderly and their family members.

*O1, O2, O3, O7, F1: “At times, healthcare professionals or individuals offering their services on a voluntary basis may conduct in-home assessments of blood pressure, blood glucose levels, and the efficacy of geriatric care, without any charge. This is precisely the requirement that I require. It is hoped that they can assist in determining the veracity of the cure.”*

*O4, O5, O6, F3, F4, F5: “It is hoped that nurses or volunteers may provide guidance for the elderly in their rehabilitation training or demonstrate the appropriate techniques to be employed. I am eager to acquire knowledge, however, I am uncertain as to whom I should seek as a source of instruction. It is uncertain whether allowing children to acquire knowledge through internet research is a reliable and credible means of obtaining information.”*

*O8, O13, O18, O19, O20, F9: “The community nurses exhibit a high level of professionalism and accountability. My spouse receives gastric tubes from community nurses. Ascending and descending stairs causes discomfort for me. I was permitted to remain seated and await assistance in navigating the formalities. Expressing gratitude to the aforementioned individuals.”*

### Theme 4: Various Factors Can Either Facilitate or Impede Interaction

#### Factors that facilitate interaction

It is anticipated that nurses will exhibit proactive and resolute behavior in providing assistance to family members during instances of illness. Collaboration is a crucial aspect of effective performance. A favorable disposition toward the cooperation among the elderly, family members, health care professionals, and volunteers has the potential to enhance social engagement. Family volunteers express a greater degree of admiration for the nurse’s interpersonal abilities in comparison to their professional competencies.

*N1: “Frequent modes of communication between my family and I include telephonic conversations and in-person visits to our residence. The present occupation appears to be intriguing as it has the potential to foster positive connections with familial caretakers and potentially streamline future professional endeavors.”*

According to certain volunteers, the involvement of community nurses in providing care for elderly individuals with chronic illnesses in low-income households is beneficial. The augmentation of nurse involvement is imperative to enhance the potential for interaction.

*N3:“Based on my observations, it appears that the responsibility of providing care for the elderly falls solely on me. Nurses exhibit infrequent involvement in the aforementioned activity, and the extent of their contribution to the service content is limited. On a weekly basis, physical examinations are conducted intermittently.”*

*N2: “It is only through the proactive pursuit of assistance that services will be rendered, albeit not always in a timely manner. The individuals in question exhibit infrequent proactivity in their attendance, and I sense a lack of familiarity with their presence.”*

Trust serves as the fundamental basis for establishing a positive relationship, and in the context of caregiving, family volunteers place their trust in the professional aptitude of a supportive community nurse for their interactions.

*F1, F3, F4:“The community nurses exhibit a high level of professionalism. It is reasonable to assume that community nurses possess a significant amount of knowledge and expertise, which enables them to effectively manage routine nursing issues.”*

Furthermore, it is imperative for community nurses and family volunteers to exhibit tolerance, minimize conflicts, prioritize the health of disabled parents as a shared objective, and actively foster collaborative relationships.

*C1, C2: “Notwithstanding, mutual comprehension and esteem are present among us, as our paramount objective is the well-being of our progenitors.”*

*C3: “Owing to variations in nursing expertise, I occasionally encounter challenges in effectively communicating with family volunteers. I possess a comprehensive understanding of the matter at hand and endeavor to communicate it to others in a calm and composed manner in order to prevent any potential disputes.”*

#### Factors that impede interaction

The interaction between community nurses and family volunteers is complex owing to factors such as demanding work schedules, time constraints, and unfavorable attitudes. Community nurses are often occupied with their routine responsibilities and may experience limitations in terms of time and resources to engage in regular communication with family volunteers of elderly individuals with disabilities. The emphasis on efficiency among community nurses is often observed to result in hasty actions, excessive focus on procedural aspects, and disregard for the preferences of family volunteers. This tendency can hinder the development of a positive interactive dynamic between community nurses and family volunteers.

*C1, N3: “A significant amount of effort is dedicated to conducting physical assessments on the elderly population, as well as providing ongoing monitoring for individuals with diabetes and hypertension. Frequently, a considerable number of family volunteers proactively reach out to me to avail of my door-to-door service. Due to my busy schedule, I am unable to engage with my family volunteers. The statement posits an impossibility.”*

The standard utilized for charging has an impact on the level of interaction. Certain volunteers express concerns regarding the elevated costs. The volunteers opt to provide care for the elderly with disabilities independently, exhibiting infrequent communication and interaction with community nurses, and declining the assistance and services offered by the aforementioned health care professionals.

*C2, N2: “The geriatric individual was confined to bed and required enteral feeding via a gastric tube. The individual's offspring procured a gastric tube via the internet in an effort to reduce costs and proceeded to self-administer its insertion. However, her level of professionalism did not match ours. However, her level of professionalism is not on par with ours. During that particular moment, she introduced the trachea, resulting in the elderly gentleman contracting a lung infection.”*

Furthermore, community nurses exhibit inadequacies in their ability to manage the health of elderly individuals with disabilities, and have not undergone training in effective communication techniques.

*C3, N1: “The equilibrium of professional caliber among community nurses was not achieved. Currently, there is a lack of specialized community nurses who are solely devoted to providing care for disabled elderly individuals and facilitating communication with their family volunteers. The presence of additional elements would be advantageous. Community nurses exhibit a limited understanding of the professional knowledge pertaining to the elderly population with disabilities. Enhancing the caliber of personnel and reinforcing training programs are imperative measures to be taken.”*

## Discussion

The aging of the population at a deeper level has resulted in a gradual and challenging problem of long-term care for chronically ill parents. Chronic illness among parents presents a complex situation where they desire to receive care at home, yet the limited capacity of family members to provide support poses a challenge. Consequently, the feasibility of providing long-term care for chronically ill parents at home is compromised, leading to an increased demand for community-based care. Community-based maintenance services and family care.^32^

It is possible to amalgamate the benefits of proficient community services and effective familial care.^33^ Several studies have indicated that establishing a congenial and amicable social milieu can foster favorable interactions among family members and the community, leading to an enhancement in the standard of care for chronically ill parents at home, and a decrease in familial and communal burdens.^34^ The research has indicated the significance of high-quality social interaction between family members and formal care providers in residential care settings. However, there is limited knowledge regarding these relationships in community-based care settings, which cater to a majority of individuals with chronic conditions.^35^

The study employed semi-structured interviews as a data collection method to elicit information from community nurses and volunteers of the elderly family chronic illness regarding their experiences and attitudes toward interaction. The present study employs a methodology of data collection and directed content analysis to identify four overarching themes, namely information interactions, emotional interactions, practical interactions, and factors that facilitate or impede interactions. The act of collaborating is deemed as a significant attribute of the interactions that occur between family volunteers and service providers who are based in the community.^36^ The establishment of collaborative and reciprocal relationships between family volunteers and community volunteers has the potential to enhance the provision of care for chronically ill parents, while simultaneously alleviating the burden experienced by family volunteers.^37^

Establishing a strong connection can enhance the collaborative partnership between family volunteers and community nurses, as evidenced by previous studies.^38^ Currently, volunteers have restricted access to support and community nursing resources, and a satisfactory collaborative relationship is absent. According to other family volunteers, community nurses frequently have restricted service time, which hinders their ability to engage in comprehensive communication with them. Consequently, the volunteers endeavor to enhance the frequency of communication with community nurses and offer diverse modes of communication to attain efficacious communication.^39^ Access professional assistance such as counseling, informational resources, and tangible aid. According to research findings, family volunteers who perceive a greater degree of support tend to exhibit a significantly greater level of contentment with regard to professional providers.^40^ Nonmedical family volunteers are seeking a community-based platform that facilitates the exchange of health-related information and counseling to enhance their knowledge and skills in caring for chronically ill parents. Regarding practical assistance, they really support assistance from any medical personnel.

Community nurses continue to face challenges in delivering services that meet the desired standards in terms of both quality and quantity. Because of the prevalence of chronic illnesses among parents, it is imperative for community nurses to conduct regular visits in order to monitor their health status over an extended period of time.^41^ Because of the health issues faced by parents with chronic illnesses, their limited maintenance skills, and the challenges posed by their volunteers, there is a desire to engage professional nurses to provide services that can alleviate their burden.^42^ Furthermore, the volunteers have raised concerns regarding challenges in accessing medical care and have expressed a desire for assistance from community nurses in facilitating access to medical professionals. Community-based service institutions tend to prioritize the quantifiable aspects of parenting, such as the duration of service and completion of tasks, over the social and emotional dimensions.^43^ The volunteers experience a significant psychological burden and cannot be relieved from prolonged periods of high psychological pressure, as evidenced by previous studies.^44^ Thus, it is imperative for volunteers to receive psychological counseling in order to mitigate the stress associated with caregiving and sustain their positive emotional well-being. Prior research has demonstrated the significance of health care providers’ assistance in promoting the mental well-being of individuals who provide care for their family members.^45^

The acknowledgment and commendation of community nurses toward the volunteers’ endeavors can enhance their self-gratification and sense of dignity, thereby facilitating superior care provision for parents afflicted with chronic illnesses. This text explores the various factors that facilitate or impede interaction between individuals. Currently, the involvement of community nurses in the care of elderly individuals with chronic illnesses residing in low-income households is limited. According to research, nurses tend to lack initiative in their interactions with family volunteers.^46^ In order to facilitate professional interactions between volunteers and community nurses, it is imperative to enhance the participation of community nurses and encourage their active involvement in the care of households with parents afflicted by chronic illnesses. Moreover, it is imperative for community nurses and family volunteers to exhibit mutual tolerance, minimize instances of discord, prioritize the health of the elderly as a shared objective, and proactively foster collaborative alliances.^47^ Nevertheless, certain community nurses exhibit inadequacy in managing the health of parents afflicted with chronic illnesses, and the caliber of community nurses’ professionalism is inconsistent. Inadequate care may be provided by certain family volunteers who exhibit poor physical conditions, low cooperation, and diminished attention. These circumstances are likely to impede the communication and collaboration between familial volunteers and community-based nurses. Various interventions have been created and evaluated to promote favorable interactions, such as support and collaboration, between family members and care providers in nursing homes.^48^ Community-based maintenance arrangements can be adapted to accommodate such interventions. According to research studies,^49^ family members tend to assume a larger portion of parenting responsibilities in community-based settings as opposed to nursing home environments.^50^

The research findings suggest that there is a need for interventions aimed at enhancing interactions among the elderly, families, volunteers, and nurses to facilitate effective collaboration and mutual support. Subsequent research endeavors can build on these implications to advance the field. Furthermore, it is imperative for governmental and social institutions to establish an appropriate setting that facilitates the enhancement of interactive relationships between the elderly, families, volunteers, and nurses. This is crucial in order to ensure the provision of superior care to elderly individuals afflicted with chronic illnesses.

## Strengths and Limitations

The present study is a qualitative investigation aimed at exploring the phenomenon of interaction among elderly individuals, their family members, nurses, and volunteers. Data are gathered from participants regarding their voting patterns and their perceptions of direct interaction. In addition to the significant contribution of this study to the implementation of the FCC approach for the treatment of a chronically disabled parent in their home, it is imperative to acknowledge two limitations. The present investigation derives its robustness from diverse interview formats and centers on the group data acquisition process to yield the disclosed outcomes. Notwithstanding the data saturation achieved through the inclusion of 36 interviews and FGD results, our findings are constrained by their qualitative nature and the exclusive focus on community arrangements. The enrichment of findings can be achieved through the collection of data from diverse settings. Certain data are gathered in a vernacular tongue and subsequently rendered into English with the aid of a language specialist, which could potentially lead to a loss of data.^51^ The post-COVID-19 adaptation has had a significant impact on our research behavior. There exist disparities in the recruitment and outreach strategies employed to engage qualified individuals.^52^ Thus, the present study is constrained by a limited sample size, thereby restricting the scope for generalization and representation. The employment of a particular sampling technique may result in the occurrence of selection and response biases.^53^ Family volunteers are selected for participation in the study using purposive sampling methodology, facilitated by health care professionals such as nurses. The acquisition of a beak ticket can be perceived as a means of fostering a stronger rapport with the nursing profession. We engage in communication with past community nurses in order to mitigate potential biases in our current responses. In certain instances, community nurses may be present during interviews, thereby increasing the likelihood of family volunteers providing a favorable assessment of their interactions with community nursing.

## Conclusion

The implementation of family-centered interventions that prioritize the collaboration between health care professionals, such as nurses and volunteers, with families, particularly those with parents experiencing chronic illness, is a crucial area of focus. Such interventions have the potential to alleviate the burden on families and improve the quality of care provided. This study offers recommendations for improving community and home care for parents with chronic diseases. The findings suggest that interventions aimed at promoting positive interactions can enhance the quality of treatment for elderly individuals with chronic illness. These insights provide a fresh perspective on how to develop and implement effective interventions in this context. It is recommended that future research employ multiple methodologies to investigate FCC across diverse health practices for the elderly population, with the aim of standardizing optimal practices and enhancing health outcomes for both elderly individuals and their families.

## Abbreviations Used

C: Nurses
D: Focus Group Discussions
F: Families
FCC: Family-Centered Care
I: Interviews
O: Older
R: Volunteers

## References

[1] Abukari AS, Acheampong AK, Aziato L. Experiences and contextual practices of family-centered care in Ghanaian Nicus: A qualitative study of families and clinicians. BMC Health Serv Res 2022;22(1):1051–1058; doi: 10.1186/s12913-022-08425-0 35978324 PMC9386929

[2] Hwang AS, Rosenberg L, Kontos P, et al. Sustaining care for a parent with dementia: An indefinite and intertwined process. Int J Qual Stud Health Well-Being 2017;12(sup2):1389578; doi: 10.1080/17482631.2017.1389578 29050539 PMC5654011

[3] Öcek ZA, Çiçeklioğlu M, Yücel U, et al. Erratum: Family medicine model in Turkey: A qualitative assessment from the perspectives of primary care workers. BMC Fam Pract 2015;16(1):74. 26113078 10.1186/s12875-015-0293-y PMC4480990

[4] Guo P, Zhang S, Niu M, et al. A qualitative study of the interaction experiences between family caregivers and community nurses for disabled elderly people at home. BMC Geriatr 2023;23(1):243; doi: 10.1186/s12877-023-03917-y 37085787 PMC10119826

[5] Hajradinovic Y, Tishelman C, Lindqvist O, et al. Family memberś experiences of the end-of-life care environments in acute care settings–a photo-elicitation study. Int J Qual Stud Health Well-Being 2018;13(1):1511767; doi: 10.1080/17482631.2018.1511767 30176152 PMC6127834

[6] Spoorenberg SLW, Wynia K, Fokkens AS, et al. Experiences of community—living older adults receiving integrated care based on the chronic care model: A qualitative study. PLoS One 2015;10(10):e0137803. 26489096 10.1371/journal.pone.0137803 PMC4619446

[7] Yakubu K, Malan Z, Colon-Gonzalez MC, et al. Perceptions about family-centred care among adult patients with chronic diseases at a general outpatient clinic in Nigeria. Afr J Prim Health Care Fam Med 2018;10(1):1739. 30456976 10.4102/phcfm.v10i1.1739 PMC6244322

[8] Rekawati E, Sari N, Istifada R. “Family support for the older person”: Assessing the perception of the older person as care recipient through the implementation of the cordial older family nursing model. Enferm Clin 2019;29(Insc 2018):205–210.

[9] Mohd Hanafiah AN, Johari MZ, Azam S. A qualitative study on the implementation of family health team: The perspectives of providers and patients. BMC Fam Pract 2020;21(1):162. 32772931 10.1186/s12875-020-01217-7 PMC7416414

[10] Neller SA, Hebdon MT, Wickens E, et al. Family caregiver experiences and needs across health conditions, relationships, and the lifespan: A Qualitative analysis. Int J Qual Stud Health Well-Being 2024;19(1):2296694; doi: 10.1080/17482631.2023.2296694 38213230 PMC10791097

[11] Jika BM, Khan HTA, Lawal M. Exploring experiences of family caregivers for older adults with chronic illness: A scoping review. Geriatr Nurs 2021;42(6):1525–1532. 34735999 10.1016/j.gerinurse.2021.10.010

[12] Ploeg J, Canesi M, Fraser KD, et al. Experiences of community-dwelling older adults living with multiple chronic conditions: A qualitative study. BMJ Open 2019;9(3):e023345. 10.1136/bmjopen-2018-023345 PMC6475239 30898800

[13] Bahtiar B, Sri Widiastuti IAK, Nopriyanto D, et al. Lived experiences constraints of family caregivers in caring for older adults with chronic diseases during the Covid-19 pandemic: A qualitative study of Indonesian perspectives. Work with Older People 2023.

[14] Teixeira MJC, Abreu W, Costa N, et al. Understanding family caregivers’ needs to support relatives with advanced progressive disease at home: An ethnographic study in rural Portugal. BMC Palliat Care 2020;19(1):73. 32450848 10.1186/s12904-020-00583-4 PMC7249372

[15] Howard AF, Crowe S, Choroszewski L, et al. When chronic critical illness is a family affair: A multi-perspective qualitative study of family involvement in long-term care. Chronic Illn 2023;19(4):804–816. 36426509 10.1177/17423953221141134 PMC10655697

[16] Zulka AN, Suryaningsih Y, Nofia P, et al. Evaluasi Manajemen Penyakit dan Psychological Well Being Lansia di Masa Pandemi Covid 19. Indones J Heal Sci 2022;14(1)

[17] Rashighi M, Harris JE. A qualitative study of factors that influence active family involvement with patient care in the ICU: Survey of critical care nurses. Physiol Behav 2017;176(3):139–148. 29169879 10.1016/j.iccn.2017.08.008 PMC5736422

[18] Shields L, Pratt J, Hunter J. Family centred care: A review of qualitative studies. J Clin Nurs 2006;15(10):1317–1323. 16968436 10.1111/j.1365-2702.2006.01433.x

[19] Doyle L, McCabe C, Keogh B, et al. An overview of the qualitative descriptive design within nursing research. J Res Nurs 2020;25(5):443–455. 34394658 10.1177/1744987119880234 PMC7932381

[20] Stolterman E. The nature of design practice and implications for interaction design research. Int J Des 2008;2(1):55–65. Available from: https://www.scopus.com/inward/record.uri?eid=2-s2.0-51149097501&partnerID=40&md5=3d1b8af02bdfa0030d5b592a6b3453a3

[21] Guest G, Bunce A, Johnson L. How many interviews are enough?: An experiment with data saturation and variability. Field Methods 2006;18(1):59–82; doi: 10.1177/1525822X05279903

[22] Braun V, Clarke V. Using thematic analysis in psychology. Qual Res Psychol 2006;3(2):77–101; doi: 10.1191/1478088706qp063oa

[23] Folstein MF, Folstein SE, McHugh PR. “Mini-mental state”. A practical method for grading the cognitive state of patients for the clinician. J Psychiatr Res 1975;12(3):189–198; doi: 10.1016/0022-3956(75)90026-6 1202204

[24] Fitriyani RA, Waluyo A. Family acceptance, peer support, and HIV serostatus disclosure of MSM-PLWHA in Medan, Indonesia. Enferm Clin 2019;29:648–652.

[25] Daly S, McGowan A, Papalambros P. Using qualitative research methods in engineering design research. In: Proceedings of the International Conference on Engineering Design, ICED; 2013. pp. 203–12. Available from: https://www.scopus.com/inward/record.uri?eid=2-s2.0-84897610172&partnerID=40&md5=160b08c53811220eee1d0d9e178ae3f2.

[26] Anwar R, Zainal Abidin S, Hasdinor Hassan O. A practical guideline to quantifying qualitative analyses of design cognition. Turkish Online J Educ Technol 2015;2015:13–21. Available from: https://www.scopus.com/inward/record.uri?eid=2-s2.0-84957537804&partnerID=40&md5=117349d93361071f99998e3ad4026962

[27] Chen F, Terken J. Analytic Methods. In: Springer Tracts in Mechanical Engineering. Springer; 2023. pp. 181–211. Available from: https://www.scopus.com/inward/record.uri?eid=2-s2.0-85136828643&doi=10.1007%2F978-981-19-3448-3_11&partnerID=40&md5=b79265a0a14713cb38d07114343cf2a9

[28] Wall J, Aeddula OK, Larsson T. Data analysis method supporting cause and effect studies in product-service system development. In: Proceedings of the Design Society: DESIGN Conference; 2020. pp. 461–70. Available from: https://www.scopus.com/inward/record.uri?eid=2-s2.0-85105908905&doi=10.1017%2Fdsd.2020.123&partnerID=40&md5=cebf3d53cffc276092d8b9717d5bc20b.

[29] Lovei P, Deckers E, Funk M, et al. The Marios and Luigis of design: Design plumbers wanted! In: DIS 2020 Companion—Companion Publication of the 2020 ACM Designing Interactive Systems Conference; 2020. pp. 197–201. Available from: https://www.scopus.com/inward/record.uri?eid=2-s2.0-85090156554&doi=10.1145%2F3393914.3395898&partnerID=40&md5=6d23640bfa6a50a67c427a73c0bc117a.

[30] Yang KL, Hsu SC, Hsu HM. Enriching design thinking with data science: Using the Taiwan moving industry as a case. In: Communications in Computer and Information Science; 2020. pp. 185–202. Available from: https://www.scopus.com/inward/record.uri?eid=2-s2.0-85080958309&doi=10.1007%2F978-981-15-3118-7_12&partnerID=40&md5=63effb116d6c9f4af01c9cb89a307d0f.

[31] Lupieri G, Creatti C, Palese A. Cardio-thoracic surgical patients’ experience on bedside nursing handovers: Findings from a qualitative study. Intensive Crit Care Nurs 2016;35:28–37; doi: 10.1016/j.iccn.2015.12.001 27080568

[32] Gab Allah AR. Challenges facing nurse managers during and beyond COVID-19 pandemic in relation to perceived organizational support. Nurs Forum 2021;56(3):539–549. 33870510 10.1111/nuf.12578 PMC8250948

[33] Tipseankhum N, Tongprateep T, Forrester DA, et al. Experiences of people with advanced cancer in home–based palliative care. Pacific Rim Int J Nurs Res 2016;20(3):238–251. Available from: http://search.ebscohost.com/login.aspx?direct=true&db=ccm&AN=116406915&site=ehost-live&scope=site

[34] Tyas MDC, Sepdianto TC, Solikhah FK, et al. Peer support education reducing pain perception and improving blood glucose control of diabetes mellitus. Indian J Public Heal Res Dev 2020;11(03):2591–2595.

[35] Buregyeya E, Kulane A, Kiguli J, et al. Motivations and concerns about adolescent tuberculosis vaccine trial participation in rural Uganda: A qualitative study. Pan Afr Med J 2015;22:76–77. 26834929 10.11604/pamj.2015.22.76.7097 PMC4725648

[36] Coleman P. Purpose, quality, and value in critical realist research within nurse education. Nurs Med J Nursing 2019;9(1):103–116.

[37] Carver H, Lazarsfeld-Jensen A. Operationalising the multidimensional role of the paramedic preceptor. Australas J Paramed 2018;15(4):1–10.

[38] Yusuf A, Hilfida NH, Krisnana I, et al. Inflence of picture and picture method against moral development of children. Ind Jour of Publ Health Rese & Develop 2018;9(10):318.

[39] Costa RRdO, Medeiros SMd, Coutinho VRD, et al. Satisfaction and self-confidence in the learning of nursing students: Randomized clinical trial. Esc Anna Nery 2020;24(1):1–9.

[40] Aditya RS, Yusuf A, Alrazeeni DM, et al. “We are Tired but Do Not Give Up” the dilemma and challenges of primary nurses facing the omicron variant: Qualitative research. J Multidiscip Healthc 2023;16(March):797–809. 37006344 10.2147/JMDH.S404177 PMC10065016

[41] Aditya RS, Yusuf A, Al Razeeni DM, et al. “We Are at The Forefront of Rural Areas” emergency nurse’s experience during pandemic: A qualitative study. Health Equity 2021;5(1):818–825. 35018314 10.1089/heq.2021.0080 PMC8742295

[42] Aditya RS, Yusuf A, Solikhah FK, et al. Nurse’s experiences in handling stretcher patients on commercial medical escort in Indonesia: A qualitative study. Bangladesh J Med Sci 2022;21(3):502–511.

[43] Yusuf A, Aditya RS, Fitryasari R, et al. Evaluation of aggressive behaviour management in PICU (Psychiatric intensive care unit): A focus group study. J Glob Pharma Technol 2020;12(6):335–339.

[44] Rahmatika QT, Aditya RS, Yusuf A, et al. We are facing some barriers: A qualitative study on the implementation of kangaroo mother care from the perspectives of healthcare providers. J Public Health Afr 2022;13(Suppl 2):2412–2463. 37497131 10.4081/jphia.2022.2412 PMC10367030

[45] Johnson-Mallard V, Jones R, Coffman M, et al. The Robert Wood Johnson nurse faculty Scholars diversity and inclusion research. Health Equity 2019;3(1):297–303. 31289788 10.1089/heq.2019.0026 PMC6608697

[46] Squires A, Dorsen C. Qualitative research in nursing and health professions regulation. J Nurs Regul 2018;9(3):15–26; doi: 10.1016/S2155-8256(18)30150-9

[47] Sepahvand F, Atashzadeh-Shoorideh F, Parvizy S, et al. Factors affecting nurses’ perceived organizational commitment: A qualitative study. Bangladesh J Med Sci 2019;18(2):303–311.

[48] Leite SCC, Caldeira AP. Therapeutic workshops and psychosocial rehabilitation for institutionalised leprosy patients. Cien Saude Colet 2015;20(6):1835–1842. 26060961 10.1590/1413-81232015206.16412014

[49] Gomes ATdL, Silva MDF, Dantas BAdS, et al. Perfil epidemiológico das emergências traumáticas assistidas por um serviço pré-hospitalar móvel de urgencia. eglobal 2016;16(1):384.

[50] Vecere A, Monteiro R, Ammann WJ, et al. Predictive models for post disaster shelter needs assessment. Int J Disaster Risk Reduct 2017;21(September 2017):44–62; doi: 10.1016/j.ijdrr.2016.11.010

[51] Jang HY. Partnership between staff and family in long-term care facility: A hybrid concept analysis. Int J Qual Stud Health Well-Being 2020;15(1):1801179. 32835642 10.1080/17482631.2020.1801179 PMC7482886

[52] Dicks SG, Northam HL, van Haren FMP, et al. The bereavement experiences of families of potential organ donors: A qualitative longitudinal case study illuminating opportunities for family care. Int J Qual Stud Health Well-Being 2023;18(1):2149100; doi: 10.1080/17482631.2022.2149100 36469685 PMC9731585

[53] Lao SSW, Le Low LP, Wong KKY. Older residents’ perceptions of family involvement in residential care. Int J Qual Stud Health Well-Being 2019;14(1):1611298; doi: 10.1080/17482631.2019.1611298 31072244 PMC6522931

